# Hypertension mediates the association between weight-adjusted waist index and new onset stroke risk in middle-aged and older Chinese adults: evidence from the CHARLS study

**DOI:** 10.3389/fneur.2025.1587176

**Published:** 2025-07-11

**Authors:** Fei Xu, Hongwei Liu, Meng Li, Minheng Zhang, Xuan Chen, Miaomiao Hou

**Affiliations:** ^1^Department of Neurology, Taiyuan City Central Hospital, The Ninth Clinical Medical College of Shanxi Medical University, Taiyuan, Shanxi, China; ^2^Department of Gerontology, The First People's Hospital of Jinzhong, Yuci, Shanxi, China; ^3^Department of Neurology, Shanxi Bethune Hospital, Shanxi Academy of Medical Sciences, Third Hospital of Shanxi Medical University, Tongji Shanxi Hospital, Taiyuan, Shanxi, China

**Keywords:** weight-adjusted waist index, stroke, pre-hypertension, hypertension, normal blood pressure

## Abstract

**Background:**

The weight-adjusted waist index (WWI) serves as a novel body metric for evaluating abdominal adiposity. However, the association between WWI and stroke risk across varying blood pressure statuses remains poorly understood.

**Methods:**

This study included 12,580 individuals aged 45 and above with no prior stroke incidents, drawn from the China Health and Retirement Longitudinal Study (CHARLS) between 2011 and 2020. Multiple analytical approaches were utilized to assess the relationship linking WWI and incident stroke, including Cox proportional hazards regression, restricted cubic splines, subgroup analyses, and sensitivity analyses. The predictive capacity of WWI for stroke was evaluated through receiver operating characteristic (ROC) curves, with further stratification based on blood pressure status and other anthropometric indicators. A mediation approach was employed to investigate the intermediary role of hypertension in the WWI-stroke association.

**Results:**

The investigation recruited 12,580 participants, 727 (5.78%) experienced stroke events. Across all Cox regression models, increased WWI consistently demonstrated a significant association with elevated stroke risk (HR = 1.12, 95%CI: 1.02–1.22, *p* = 0.012), particularly in individuals with pre-hypertension (HR = 1.20, 95% CI: 1.00–1.46, *p* = 0.044) and hypertension (HR = 1.13, 95% CI: 1.01–1.25, *p* = 0.032). Additional sensitivity examinations further corroborated the consistency of our results regarding WWI’s predictive accuracy for stroke risk. The ROC curves revealed that WWI exhibited the highest predictive accuracy for stroke risk (AUC = 0.679) among the anthropometric indices evaluated, outperforming other conventional indicators. The predictive performance was significantly stronger in the hypertension group (AUC = 0.672) than in different subgroups. Mediation effect analysis indicated that hypertension partially mediated the impact of WWI on stroke (*β*: 0.015, *p* < 0.001), accounting for 12% of WWI’s total effect on stroke incidence.

**Conclusion:**

This study revealed that elevated WWI levels independently contribute to a higher stroke occurrence rate in middle-aged and older adult demographics, with a particularly pronounced effect observed in hypertensive individuals.

## Introduction

Stroke, an acute cerebrovascular condition with complex and multifactorial origins, is a pressing global health issue. Recent epidemiological data highlight its status as the leading cause of death in China and the second most common cause of mortality worldwide (1). The aging global population has contributed to a significant rise in stroke incidence (2), with its substantial burden marked by high rates of morbidity, mortality, and disability, creating profound challenges for healthcare systems and socioeconomic stability (3).

Body mass index (BMI) and waist circumference (WC) represent the most widely utilized indices for assessing obesity. While BMI exhibits significant variability across different demographic parameters including age, gender, and ethnic background (4). WC demonstrates a stronger association with visceral adipose tissue accumulation (5). The clinical utility of WC as an independent obesity indicator is constrained by its inherent correlation with BMI. To address this limitation, the weight-adjusted waist index (WWI) has been developed as an innovative metric that integrates the advantages of WC measurements while maintaining consistent associations with abdominal adiposity (6). This index, computed by dividing the waist circumference by the square root of the body weight, effectively addresses the correlation between BMI and WC by specifically assessing central obesity independent of overall body mass. Growing epidemiological evidence indicates that these anthropometric measures are significantly associated with higher incidence rates of hypertension, cerebrovascular events, and various cardiovascular diseases (7, 8). Recent studies in Chinese populations have further revealed that elevated WWI levels are independently linked to increased risks of both all-cause mortality and cardiovascular-related deaths. As a result, WWI has emerged as a valuable predictive tool for adverse health outcomes in clinical practice, particularly for cardiovascular risk assessment and mortality prediction.

The development of stroke involves multiple mechanisms, with obesity being a key contributing factor (9). While the previously published study did examine the association between the WWI and stroke risk, its analysis was relatively straightforward and did not account for important contextual factors (10). In contrast, our study specifically investigates the effect of WWI on stroke risk across different blood pressure categories, recognizing that hypertension remains the leading modifiable risk factor for stroke. By stratifying participants into normotensive, prehypertensive, and hypertensive subgroups, we assessed whether the association between WWI and stroke risk varied according to blood pressure status. Should such variation be confirmed, it would provide evidence to further examine whether hypertension mediates the relationship between WWI and incident stroke risk within the China Health and Retirement Longitudinal Study (CHARLS) cohort. Moreover, considering that hypertension is a widely acknowledged and alterable contributor to stroke risk (11), and earlier research has established a significant connection between WWI and the onset of hypertension (12, 13), is possible that hypertension functions as a link between WWI and stroke. Analyzing this potential mediation pathway could offer new perspectives on the pathophysiological mechanisms that connect central adiposity to cerebrovascular outcomes. Given the well-established pathophysiological links among hypertension, central adiposity, and cerebrovascular events, along with the pressing need for a comprehensive understanding of modifiable risk factors to inform preventive strategies, our study leverages CHARLS, a nationally representative cohort with standardized anthropometric and clinical assessments. Using this dataset, we conduct a stratified analysis to elucidate the association between WWI and stroke risk across blood pressure categories. Additionally, we explore the potential mediating role of hypertension in the relationship between WWI and incident stroke.

## Methods

### Study design and participants

**Figure 1:**
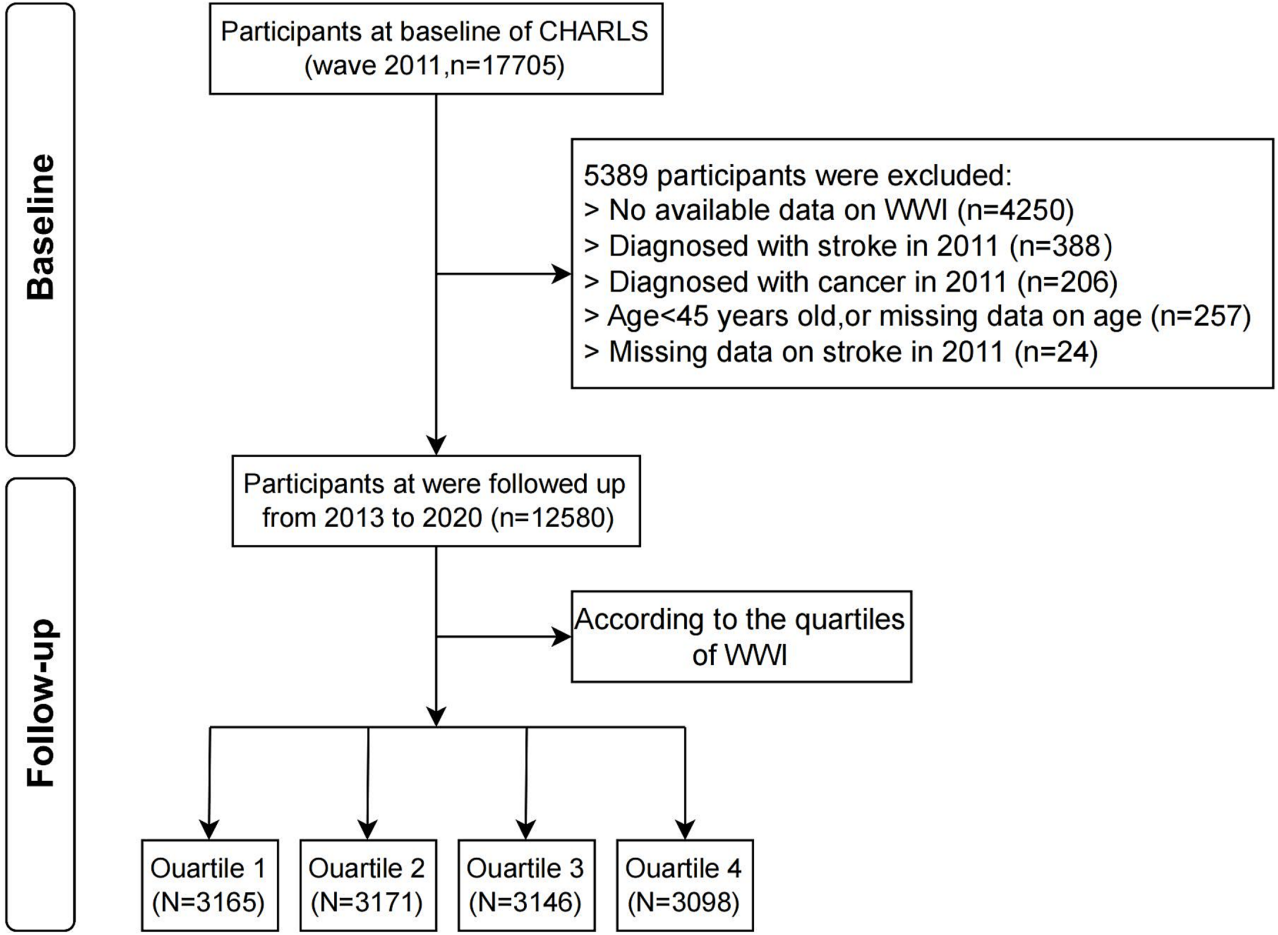
Flowchart illustrating the selection process of study participants. WWI, weight-adjusted waist index; CHARLS, China Health and Retirement Longitudinal Study.

This investigation is based on a secondary analysis of data from the CHARLS, which is publicly available^1^. CHARLS is a longitudinal survey conducted nationwide, focusing on Chinese adults aged 45 and older, and employs a stratified multistage probability sampling method covering 150 county and district-level units in 28 provinces of China. Detailed information on demographic attributes, health status, socioeconomic factors, and retirement behaviors is included in the dataset (14). Over the period from 2011 to 2020, data were collected biennially to triennially, completing five survey waves. At baseline (2011), 17,705 subjects were enrolled, with 5,125 individuals excluded from the analysis based on predefined exclusion criteria: (1) no available data on WWI; (2) diagnosed with stroke in 2011; (3) diagnosed with cancer in 2011; (4) age<45 years old, or missing data on age; (5) missing data on stroke in 2011. Following these exclusions, the remaining 12,580 eligible participants were stratified into quartiles according to their baseline WWI measurements and prospectively monitored through 2020 (Figure 1). The investigation employed de-identified variables from the publicly accessible CHARLS dataset and did not involve collecting any primary data. The Institutional Review Board of Peking University approved the study protocol, and all participants provided written informed consent before enrolling in the research.

### Calculation of weight-adjusted-waist index, waist circumference and waist-to-hip ratio

Weight-adjusted waist index (WWI) represents an innovative anthropometric indicator that specifically reflects abdominal adiposity independent of body weight (15). This is determined by taking the waist circumference in centimeters and dividing it by the square root of the body weight in kilograms. Standardized procedures were used to measure waist circumference (WC) in centimeters, while the waist-to-hip ratio (WHR) was calculated as the WC divided by hip circumference, regarded as a unitless measure. Using standardized procedures, WC was measured. The measurement of hip circumference was taken at the broadest section of the buttocks using a tape that does not stretch, with the participant standing and feet together. The waist-to-hip ratio (WHR) is calculated as the waist circumference divided by the hip circumference and is treated as a variable without units. Body weight was quantified using calibrated digital scales, with participants required to wear standardized examination gowns and maintain a standardized standing position at the center of the scale, arms positioned naturally at the sides, and gaze directed forward (14). The mobile examination center had certified health technicians measure participants’ weight and waist circumference. To measure participants’ weights, shoes and heavy clothing were removed, and WC was assessed by marking a horizontal line above the highest lateral edge of the right iliac bone to align with the right mid-axillary line and using a tape measure at the intersection of the two lines (16). All measurements followed strict protocols to maintain consistency and reliability across the study population.

### Stroke event ascertainment

Incident stroke cases were identified among participants who were stroke-free at baseline but reported a new stroke diagnosis during follow-up assessments. A standardized, physician-validated questionnaire was administered to collect stroke-related data, including diagnostic confirmation by medical professionals. The evaluation of self-reported stroke was conducted using these questions: “Have you received a stroke diagnosis from a doctor?”; “Since the last follow-up, have you been told by a doctor that you have had a stroke?”; “How does your current stroke condition compare to the last time we spoke: better, the same, or worse?.” Stroke event timing was ascertained through participants’ responses to queries like: “When did you first know you had a stroke?”; “When did your most recent stroke take place?.” From 2011 to either the onset of a stroke or the year 2020, whichever was earlier, all participants were tracked through five-wave interviews. The instrument captured detailed information on the date of diagnosis or initial symptom onset, along with documentation of any stroke-specific therapeutic interventions (14, 17). The date of first-reported stroke diagnosis served as the index date for incidence calculation. To determine the temporal relationship of stroke events, the interval between baseline assessment and reported onset date was computed. For participants without reported stroke events during the study period, follow-up duration was calculated from baseline evaluation to the date of their last available assessment (14).

### Data collection and operational definitions

Demographic and clinical information was gathered using structured questionnaires administered by trained interviewers. The assessment encompassed basic demographic characteristics (age, gender, marital status), lifestyle factors (smoking status, alcohol consumption patterns), and medical history (hypertension and diabetes mellitus). Blood pressure measurements were obtained following a standardized protocol: after a 15-min rest period, three consecutive readings were taken from the left arm at 45-s intervals, except for participants with arm injuries. Venous blood samples were collected by medically trained personnel following strict standardized procedures for subsequent biochemical analysis. While approximately 8% of participants reported fasting durations of less than 8 h before blood collection, all samples were processed using standardized laboratory methods: fasting plasma glucose (FPG) and lipid profiles were determined through enzymatic colorimetric assays, and glycated hemoglobin A1c (HbA1c) levels were quantified using boronate affinity high-performance liquid chromatography (18).

Covariate selection was informed by extant literature and clinical expertise. The covariates encompassed demographic and clinical factors, including age, sex, marital status, educational attainment, smoking status, alcohol intake, dyslipidemia, body mass index (BMI), waist circumference (WC), waist-to-hip ratio (WHR), obesity, diabetes, hypertension, systolic blood pressure (SBP), diastolic blood pressure (DBP), heart rate, HbA1c, fasting blood glucose (FPG), creatinine, blood urea nitrogen (BUN), and estimated glomerular filtration rate (eGFR). Diabetes was ascertained based on the following criteria: FPG ≥ 126 mg/dL or HbA1c ≥ 6.5%, and/or a self-reported physician diagnosis, and/or use of antidiabetic pharmacotherapy (19). Dyslipidemia was defined by self-reported physician diagnosis, current utilization of lipid-lowering agents, and/or biochemical parameters, specifically triglyceride (TG) > 150 mg/dL, high-density lipoprotein cholesterol (HDL-C) < 40 mg/dL, low-density lipoprotein cholesterol (LDL-C) > 160 mg/dL, or total cholesterol (TC) > 240 mg/dL (20). Chronic kidney disease was operationalized as a self-reported physician diagnosis or estimated eGFR<60 mL/min/1.73m^2^, in accordance with the methodology employed in the CHARLS studies (21). Hypertension was defined as a self-reported physician diagnosis, use of antihypertensive medications, and/or an average SBP/DBP ≥ 140/90 mmHg (22).

### Statistical analysis

Statistical analyses were performed utilizing R software (version 4.4.1), with a predefined significance threshold of *p* < 0.05. The initial distributional checks revealed that continuous variables were normally distributed, so they were summarized using means and standard deviations. In contrast, categorical variables were expressed as absolute frequencies and relative percentages. Intergroup comparisons were executed employing the Kruskal–Wallis rank-sum test for non-parametric continuous data and Pearson’s chi-square test for categorical variables. The issue of missing data was addressed through the application of multiple imputations via chained equations (MICE), utilizing the mice package within the R statistical environment (Supplementary Table 1).

In the analytical framework of Cox proportional hazards regression, the WWI variable was evaluated both as a continuous metric and as a categorical variable segmented by quartile intervals. For stratification, WWI was divided into quartiles: 3.34 ≤ Q1 ≤ 10.59, 10.59 < Q2 ≤ 11.13, 11.13 < Q3 ≤ 11.72, and 11.72 < Q4 ≤ 17.89, with Q1 serving as the reference group. The relationship between WWI and stroke incidence was assessed using three Cox proportional hazards regression models. Selection of covariates was guided by previous studies and their relevance to clinical practice. The model’s validity was supported as the proportional hazards assumption showed no significant violations when evaluated with Schoenfeld residuals (*p* > 0.05 for all covariates). To assess potential multicollinearity, variance inflation factors were calculated, with all values being less than 3, indicating no major collinearity among the covariates. By comparing alternative model specifications with the Akaike Information Criterion (AIC) and Bayesian Information Criterion (BIC), we selected the model that achieved the best trade-off between complexity and fit. Model 1 provided unadjusted estimates of WWI’s impact on stroke risk. Model 2 accounted for essential demographic and lifestyle variables such as age, gender, education level, marital status, smoking status, and drinking status, whereas Model 3 further incorporated clinical and laboratory variables such as creatinine, BUN, eGFR, diabetes, dyslipidemia, and hypertension. Subgroup analyses examined the relationship between WWI and stroke risk across age groups (<60 and ≥60 years), sex, married and living with spouse, drinking status, smoking status, diabetes, dyslipidemia, and hypertension status, while incorporating all potential confounding variables through multivariate adjustment (Model 3). Furthermore, the relationship between WWI and stroke was analyzed across different blood pressure states, stratified by the following factors: gender, age, marital status, smoking status, alcohol consumption, dyslipidemia, and diabetes. A forest plot was constructed to visualize these associations. To explore the potential non-linear relationship between WWI and stroke risk, a restricted cubic spline (RCS) function was employed. Four knots were selected based on the AIC minimization principle. The analysis was adjusted for all aforementioned confounding factors, and the RCS curve was plotted to illustrate the relationship. The model’s discriminative power was evaluated by generating receiver operating characteristic (ROC) curves and determining the area under the curve (AUC). An AUC value exceeding 0.6 was considered indicative of good discriminative ability. In the ROC analysis set up to evaluate the capacity to discriminate incident stroke, WWI, WC (measured in centimeters), and WHR (a dimensionless index) were incorporated as continuous predictors without any transformation. By computing the area under the ROC curve (AUC), each indicator’s ability to identify individuals who would later have stroke events was quantified, allowing for a direct comparison of their predictive performances. The discriminative performance of WWI, WC, and WHR for stroke risk prediction was compared through ROC analyses using the “pROC package.” The package facilitated the estimation of the AUC standard errors (AUC. SE), 95% confidence intervals (AUC.low and AUC.up), and *p*-values to analyze the predictive capability of WWI, WC, and WHR for stroke risk. In addition, ROC analyses generated supplementary performance metrics, including the optimal cutoff value, overall accuracy (ACC), sensitivity (SEN), specificity (SPE), positive likelihood ratio (PLR), negative likelihood ratio (NLR), positive predictive value (PPV), negative predictive value (NPV), positive predictive agreement (PPA), negative predictive agreement (NPA), total predictive agreement (TPA), and the kappa statistic (KAPPA), each with their respective 95% confidence intervals. To investigate whether hypertension mediates the association between WWI and stroke, a multiple mediation effect analysis was conducted using the “mediation” package. The simulation frequency was set to 1,000 iterations to ensure a robust estimation of the mediation effects. To ensure the reliability and consistency of the results, the following sensitivity analyses were performed: The association between WWI and the risk of stroke was reanalyzed using datasets with imputed missing values to account for potential bias. To address potential reverse causality, the analysis was repeated after excluding participants who experienced a stroke within the first 2 years of cohort entry.

## Results

### Participant demographics and characteristics

Table 1 delineates the baseline profiles of 12,580 eligible subjects categorized by WWI quartiles. The analytical sample demonstrated balanced gender representation (male: 5,973, 47.48%; female: 6,607, 52.52%). Progressive age increments across ascending WWI quartiles were evident: Q1 (mean 56.72 years, SD 8.58), Q2 (57.67 years, SD 8.95), Q3 (59.56 years, SD 9.25), and Q4 (64.21 years, SD 9.95). Comparative analysis of quartile strata demonstrated significant heterogeneity (*p* < 0.001) in three domains: (1) sociodemographic parameters: Age distribution, gender composition, marital status, and educational attainment; (2) lifestyle/metabolic factors: Smoking behavior, alcohol consumption, adiposity indices (BMI, WC, WHR, obesity status), glycemic markers (HbA1c, FPG), and cardiometabolic comorbidities (dyslipidemia, diabetes, hypertension); (3) Hemodynamic/renal profiles: Blood pressure parameters (SBP, DBP), heart rate, and renal function indicators (creatinine, eGFR). Notably, BUN levels showed no significant interquartile disparity (*p* = 0.282).

**Table 1:** Baseline population characteristics of categorized by WWI quartiles.

Characteristic	Overall	Quartile of WWI				*p*-value
		Q1	Q2	Q3	Q4	
	*N* = 12,580	*N* = 3,165	*N* = 3,171	*N* = 3,146	*N* = 3,098	
WWI, Mean ± SD	11.18 (0.97)	10.07 (0.70)	10.87 (0.16)	11.41 (0.17)	12.37 (0.62)	<0.001
Age, years, Mean ± SD	59.54 (9.64)	56.72 (8.58)	57.67 (8.95)	59.56 (9.25)	64.21 (9.95)	<0.001
BMI, kg/m^2^, Mean ± SD	23.45 (3.93)	22.16 (4.18)	23.24 (3.30)	24.10 (3.68)	24.31 (4.13)	<0.001
WC, cm, Mean ± SD	85.08 (10.31)	76.57 (7.98)	83.74 (7.84)	87.78 (8.70)	92.41 (9.48)	<0.001
WHR, Mean ± SD	0.54 (0.07)	0.47 (0.04)	0.52 (0.04)	0.56 (0.04)	0.61 (0.06)	<0.001
SBP, mmHg, Mean ± SD	131.18 (24.18)	126.04 (20.21)	129.68 (24.28)	131.74 (23.42)	137.27 (26.95)	<0.001
DBP, mmHg, Mean ± SD	75.99 (12.23)	74.79 (12.04)	76.06 (12.45)	76.37 (12.10)	76.75 (12.25)	<0.001
Heart rate, beats/min, Mean ± SD	72.81 (10.84)	71.30 (10.49)	72.37 (10.69)	73.36 (10.83)	74.22 (11.12)	<0.001
HbA1c, %, Mean ± SD	5.25 (0.75)	5.14 (0.58)	5.22 (0.75)	5.26 (0.73)	5.37 (0.89)	<0.001
FBG, mg/dl, Mean ± SD	108.05 (31.91)	104.37 (26.74)	107.28 (30.33)	109.03 (30.92)	111.54 (38.15)	<0.001
Creatinine, mg/dl, Mean ± SD	0.78 (0.21)	0.80 (0.17)	0.80 (0.20)	0.78 (0.27)	0.75 (0.20)	<0.001
BUN, mg/dl, Mean ± SD	15.60 (4.43)	15.65 (4.39)	15.69 (4.51)	15.47 (4.43)	15.57 (4.39)	0.282
eGFR, ml/min/1.73 m^2^, Mean ± SD	91.96 (15.26)	95.34 (14.07)	93.92 (15.10)	91.38 (14.93)	87.20 (15.65)	<0.001
Gender, *n* (%)						<0.001
Male	5,973.00 (47.48%)	2,163.00 (68.34%)	1,866.00 (58.85%)	1,323.00 (42.05%)	621.00 (20.05%)	
Female	6,607.00 (52.52%)	1,002.00 (31.66%)	1,305.00 (41.15%)	1,823.00 (57.95%)	2,477.00 (79.95%)	
Marital status, *n* (%)						<0.001
Married and living with a spouse	10,913.00 (86.75%)	2,849.00 (90.02%)	2,855.00 (90.03%)	2,766.00 (87.92%)	2,443.00 (78.86%)	
Other	1,667.00 (13.25%)	316.00 (9.98%)	316.00 (9.97%)	380.00 (12.08%)	655.00 (21.14%)	
Education level, *n* (%)						<0.001
Junior high school and below	8,705.00 (69.20%)	1,915.00 (60.51%)	1,980.00 (62.44%)	2,243.00 (71.30%)	2,567.00 (82.86%)	
Senior high school	3,659.00 (29.09%)	1,171.00 (37.00%)	1,122.00 (35.38%)	862.00 (27.40%)	504.00 (16.27%)	
College and above	216.00 (1.72%)	79.00 (2.50%)	69.00 (2.18%)	41.00 (1.30%)	27.00 (0.87%)	
Smoking status, *n* (%)						<0.001
Never smoked	7,585.00 (60.29%)	1,436.00 (45.37%)	1,700.00 (53.61%)	2,022.00 (64.27%)	2,427.00 (78.34%)	
Currently smoking	3,922.00 (31.18%)	1,449.00 (45.78%)	1,159.00 (36.55%)	831.00 (26.41%)	483.00 (15.59%)	
Ever smoked	1,073.00 (8.53%)	280.00 (8.85%)	312.00 (9.84%)	293.00 (9.31%)	188.00 (6.07%)	
Drinking status, *n* (%)						<0.001
Never drink	8,823.00 (70.14%)	1,975.00 (62.40%)	2,031.00 (64.05%)	2,264.00 (71.96%)	2,553.00 (82.41%)	
Currently drinking	3,143.00 (24.98%)	1,037.00 (32.76%)	959.00 (30.24%)	734.00 (23.33%)	413.00 (13.33%)	
Ever drunk	614.00 (4.88%)	153.00 (4.83%)	181.00 (5.71%)	148.00 (4.70%)	132.00 (4.26%)	
Obesity, *n* (%)						<0.001
Normal weight	7,603.00 (60.44%)	2,507.00 (79.21%)	1,958.00 (61.75%)	1,612.00 (51.24%)	1,526.00 (49.26%)	
Overweight	3,599.00 (28.61%)	525.00 (16.59%)	965.00 (30.43%)	1,073.00 (34.11%)	1,036.00 (33.44%)	
Obesity	1,378.00 (10.95%)	133.00 (4.20%)	248.00 (7.82%)	461.00 (14.65%)	536.00 (17.30%)	
Diabetes mellitus, *n* (%)						<0.001
No	11,061.00 (87.93%)	2,945.00 (93.05%)	2,817.00 (88.84%)	2,706.00 (86.01%)	2,593.00 (83.70%)	
Yes	1,519.00 (12.07%)	220.00 (6.95%)	354.00 (11.16%)	440.00 (13.99%)	505.00 (16.30%)	
Dyslipidemia, *n* (%)						<0.001
No	11,447.00 (90.99%)	2,965.00 (93.68%)	2,898.00 (91.39%)	2,817.00 (89.54%)	2,767.00 (89.32%)	
Yes	1,133.00 (9.01%)	200.00 (6.32%)	273.00 (8.61%)	329.00 (10.46%)	331.00 (10.68%)	
Hypertension, *n* (%)						<0.001
No	7,532.00 (59.87%)	2,289.00 (72.32%)	1,983.00 (62.54%)	1,808.00 (57.47%)	1,452.00 (46.87%)	
Yes	5,048.00 (40.13%)	876.00 (27.68%)	1,188.00 (37.46%)	1,338.00 (42.53%)	1,646.00 (53.13%)	
Stroke incidence, *n* (%)						<0.001
No	11,853.00 (94.22%)	3,031.00 (95.77%)	2,993.00 (94.39%)	2,959.00 (94.06%)	2,870.00 (92.64%)	
Yes	727.00 (5.78%)	134.00 (4.23%)	178.00 (5.61%)	187.00 (5.94%)	228.00 (7.36%)	

WWI, weight-adjusted waist index; SBP, systolic blood pressure; DBP, diastolic blood pressure; BMI, body mass index; WC, waist circumference; WHR, waist-to-hip ratio; HbA1c, hemoglobin A1c; eGFR, estimated glomerular filtration rate; FBG, Fasting blood glucose; BUN, blood urea nitrogen; *P*-value, Pearson’s Chi-squared test; Kruskal–Wallis rank sum test.

### WWI and incidence of stroke

Over a median follow-up duration of 8.72 years, stroke events were documented in 727 participants, accounting for 5.78% of the total study population. The stroke incidence showed a significant upward trend with increasing WWI, reaching its peak at 7.36% in the highest quartile (Q4), in contrast to 4.23% observed in the lowest quartile (Q1) (*p* < 0.001), as detailed in Table 1. Cox proportional hazards regression models consistently demonstrated a robust positive correlation between WWI elevation and stroke risk. The analysis indicated that progressive increases in WWI were significantly associated with elevated stroke incidence. Specifically, Model I revealed that each unit increase in WWI was associated with a 27% higher risk of stroke (HR = 1.27, 95%CI: 1.18–1.37, *p* < 0.001), as shown in Table 2. After additional adjustments, Model II demonstrated a slightly attenuated yet still significant association, with each unit increase in WWI corresponding to a 20% higher risk of stroke. The most comprehensive Model III, incorporating multiple covariates, maintained this significant association, demonstrating a 12% elevation in stroke risk for each unit increase in WWI (HR = 1.12, 95%CI: 1.02–1.22, *p* = 0.012), as shown in Table 2.

**Table 2:** Multivariate-adjusted HR (95% Cl) of WWI for incident stroke in three models.

Characteristic	*N*	Event *N*	Model 1			Model 2			Model 3		
			HR	95% CI	*p*-value	HR	95% CI	*p*-value	HR	95% CI	*p*-value
Total											
Continues WWI	12,580	727	1.27	1.18, 1.37	<0.001	1.20	1.10, 1.31	<0.001	1.12	1.02, 1.22	0.012
WWI (Quartile)	12,580	727									
Q1	3,165		Reference	Reference		Reference	Reference		Reference	Reference	
Q2	3,171		1.34	1.07, 1.67	0.011	1.31	1.04, 1.64	0.020	1.19	0.95, 1.49	0.127
Q3	3,146		1.41	1.13, 1.77	0.002	1.33	1.06, 1.67	0.015	1.14	0.90, 1.43	0.269
Q4	3,098		1.76	1.43, 2.18	<0.001	1.52	1.20, 1.93	<0.001	1.23	0.97, 1.57	0.088

Model 1: Unadjusted.

Model 2: Adjusted for age, gender, education level, marriage status, drinking status, smoking status.

Model 3: Model 2 with additional adjustment for creatinine, BUN, eGFR, diabetes mellitus, dyslipidemia, and hypertension.

WWI, weight-adjusted waist index; BUN, blood urea nitrogen; eGFR, estimated glomerular filtration rate; HR, hazard ratio, CI, confidence interval.

**Figure 2:**
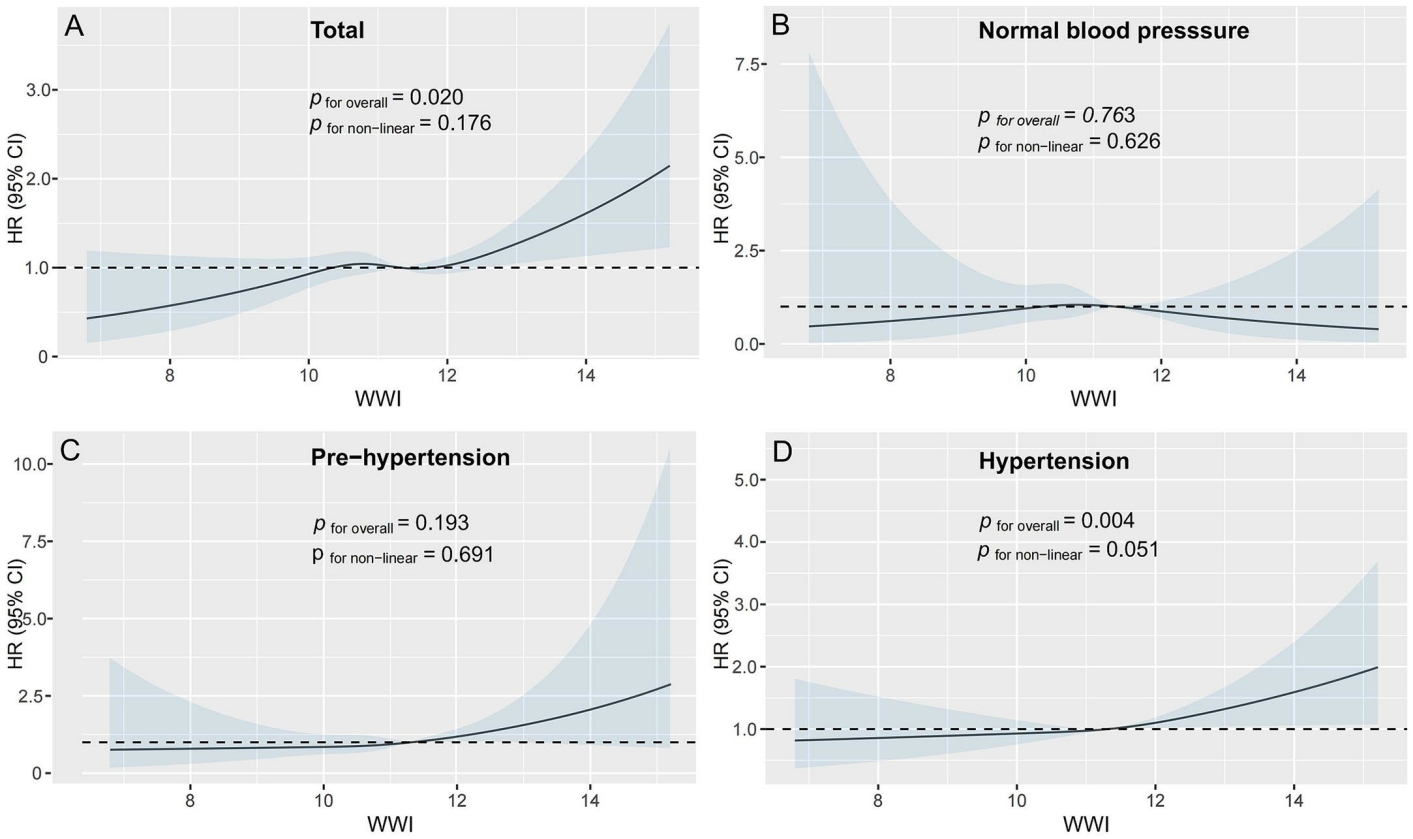
Association of WWI and the risk of stroke using a multivariable-adjusted restricted cubic spines model. **(A)** Total participants; **(B)** participants with normal blood pressure; **(C)** participants with pre-hypertension; **(D)** participants with hypertension. Model 1: Unadjusted. Model 2: Adjusted for age, gender, education level, marriage status, drinking status, smoking status. Model 3: Model 2 with additional adjustment for creatinine, BUN, eGFR, diabetes mellitus, dyslipidemia, and hypertension. WWI, weight-adjusted waist index; BUN, blood urea nitrogen; eGFR, estimated glomerular filtration rate; HR, hazard ratio, CI, confidence interval.

Figure 2A illustrates the dose–response relationship between WWI and stroke risk through RCS analysis. The RCS curve demonstrates a significant linear correlation between WWI and the probability of stroke occurrence. This linear association persists consistently across multiple adjustment models (overall: *p* = 0.020; non-linear: *p* = 0.176). The graphical representation confirms a progressive increase in stroke risk with higher WWI values.

### Blood pressure variability modifies WWI-stroke association

During follow-up, incident stroke cases were identified in 110 normal blood pressure individuals, 162 pre-hypertensive individuals, and 455 hypertensive individuals. In the pre-hypertensive cohort, full multivariable adjustment (Model III) maintained statistical significance, demonstrating a 20% incremental risk per WWI unit (HR = 1.20, 95%CI: 1.00–1.46, *p* = 0.044) (Table 3). Our analysis revealed a notable elevation in stroke risk within the highest quartile (Q4) relative to the lowest quartile (Q1) among pre-hypertensive subjects in Model I (HR = 1.75, 95%CI: 1.14–2.67, *p* = 0.010) (Table 3). In hypertensive participants, each unit in WWI correlated with 13% augmented stroke risk in Models III (HR = 1.13, 95%CI: 1.01–1.25, *p* = 0.032). However, no substantial associations were detected when comparing higher quartiles to the lowest quartile (Q1). Additionally, in the fully adjusted Model III, no significant associations were observed between WWI, whether analyzed as a continuous variable or categorized into quartiles, and stroke risk among individuals with normal blood pressure (*p* > 0.05) (Table 3). The RCS curves indicated a significant linear association between WWI and stroke incidence in hypertensive individuals, even after covariate adjustment (overall *p* = 0.004, non-linear *p* = 0.051) (Figure 2D). We visually observed a linear association between WWI and stroke incidence in the prehypertension cohort, though failed to reach conventional significance thresholds (overall *p* = 0.193, non-linear *p* = 0.691) (Figure 2C). Similarly, no significant dose–response relationship was observed between WWI and stroke risk in normal blood pressure individuals (overall *p* = 0.763, non-linearity *p* = 0.626) (Figure 2B).

**Table 3:** Association between WWI and the risk of stroke according to different blood pressure status.

Characteristic	*N*	Event *N*	Model 1			Model 2			Model 3		
			HR	95% CI	*p*-value	HR	95% CI	*p*-value	HR	95% CI	*p*-value
Normal blood pressure											
Continues-WWI	3,887	110	1.13	0.91, 1.40	0.285	0.97	0.77, 1.23	0.815	0.95	0.75, 1.20	0.659
WWI (Quartile)	3,887	110									
Q1	1,246		Reference	Reference		Reference	Reference		Reference	Reference	
Q2	1,057		1.34	0.82, 2.19	0.240	1.24	0.76, 2.04	0.394	1.21	0.74, 1.99	0.450
Q3	894		1.16	0.68, 1.98	0.578	0.97	0.56, 1.69	0.915	0.90	0.52, 1.57	0.713
Q4	690		1.27	0.73, 2.21	0.406	0.85	0.46, 1.57	0.601	0.80	0.43, 1.49	0.484
Pre-hypertension											
Continues-WWI	3,639	162	1.28	1.09, 1.52	0.004	1.22	1.01, 1.47	0.015	1.20	1.00, 1.46	0.044
WWI (Quartile)	3,639	162									
Q1	1,040		Reference	Reference		Reference	Reference		Reference	Reference	
Q2	925		0.92	0.57, 1.47	0.722	0.89	0.55, 1.44	0.643	0.88	0.54, 1.41	0.586
Q3	913		1.35	0.88, 2.08	0.169	1.30	0.83, 2.02	0.255	1.26	0.81, 1.98	0.305
Q4	761		1.75	1.14, 2.67	0.010	1.61	1.00, 2.60	0.052	1.57	0.97, 2.54	0.067
Hypertension											
Continues-WWI	5,054	455	1.13	1.03, 1.24	0.010	1.14	1.03, 1.27	0.015	1.13	1.01, 1.25	0.032
WWI (Quartile)	5,054	455									
Q1	879		Reference	Reference		Reference	Reference		Reference	Reference	
Q2	1,189		1.28	0.95, 1.74	0.110	1.31	0.96, 1.77	0.087	1.29	0.95, 1.75	0.102
Q3	1,339		1.17	0.87, 1.58	0.307	1.16	0.85, 1.58	0.341	1.11	0.81, 1.51	0.522
Q4	1,647		1.30	0.97, 1.73	0.074	1.30	0.95, 1.78	0.106	1.22	0.89, 1.67	0.223

Model 1: Unadjusted.

Model 2: Adjusted for age, gender, education level, marriage status, drinking status, smoking status.

Model 3: Model 2 with additional adjustment for creatinine, BUN, eGFR, diabetes mellitus and dyslipidemia.

WWI, weight-adjusted waist index; BUN, blood urea nitrogen; eGFR, estimated glomerular filtration rate; HR, hazard ratio, CI, confidence interval.

### Subgroup analyses

**Figure 3:**
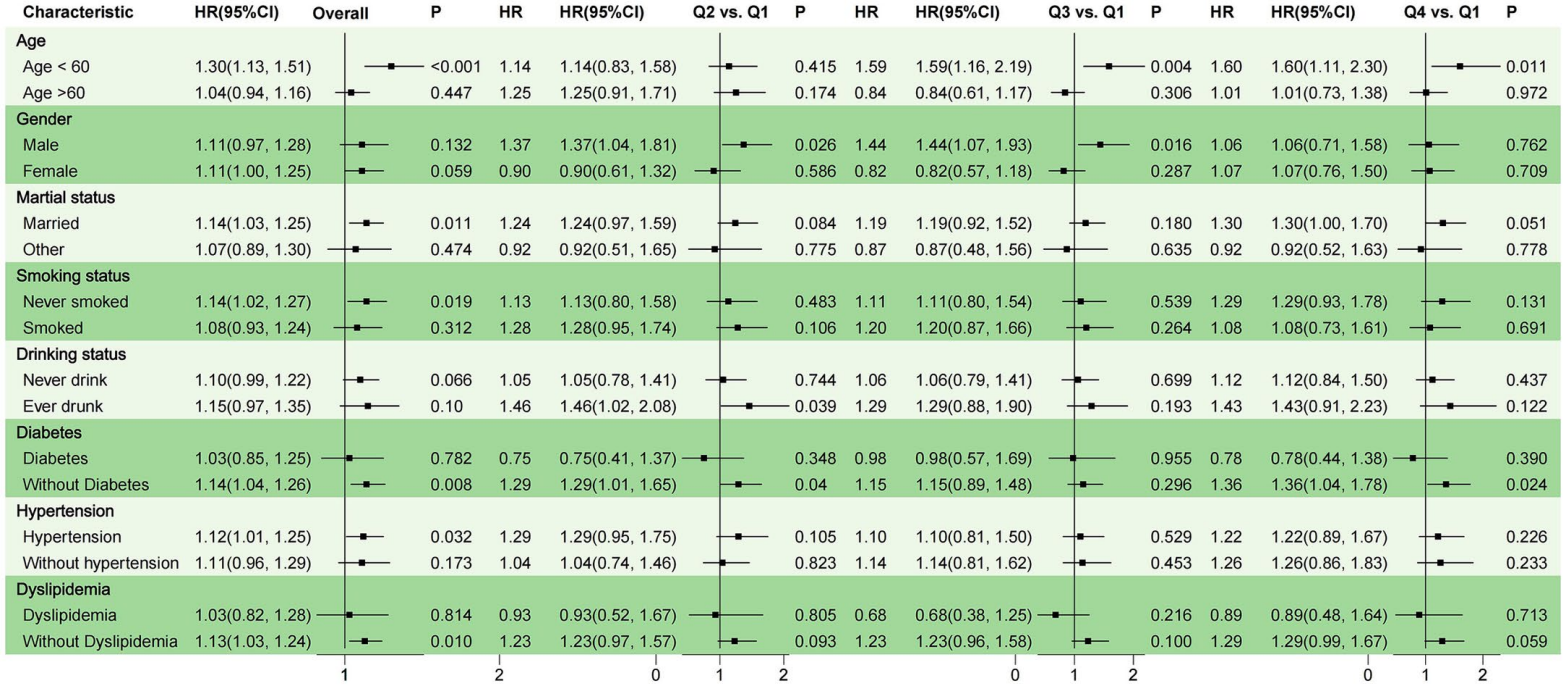
Stratified analysis of WWI and risk of stroke. Model 1: Unadjusted. Model 2: Adjusted for age, gender, education level, marriage status, drinking status, smoking status. Model 3: Model 2 with additional adjustment for creatinine, BUN, eGFR, diabetes mellitus, dyslipidemia, and hypertension. WWI, weight-adjusted waist index; BUN, blood urea nitrogen; eGFR, estimated glomerular filtration rate; HR, hazard ratio, CI, confidence interval.

The research utilized subgroup analyses to explore the link between WWI and overall incident stroke, categorized by possible risk factors, with all findings derived from Model 3. Participants were categorized depending on factors such as age, gender, marital status, smoking status, drinking status, diabetes, hypertension, and dyslipidemia (Figure 3). Individuals under the age of 60 were linked to an increased risk ratio for stroke (HR = 1.30, 95%CI: 1.13–1.51, *p* < 0.001). For individuals aged 60 and above, no significant relationship was observed (HR = 1.04, 95%CI: 0.94–1.16, *p* = 0.447). No significant difference in stroke incidence was observed between males and females overall (HR = 1.11, 95%CI: 0.97–1.28, *p* = 0.132 for males; HR = 1.11, 95%CI: 1.00–1.25, *p* = 0.059 for females). Married individuals (HR = 1.14, 95%CI: 1.03–1.25, *p* = 0.011), non-smokers (HR = 1.14, 95%CI: 1.02–1.27, *p* = 0.019), and those with hypertension (HR = 1.12, 95%CI: 1.01–1.25, *p* = 0.032) exhibit a significantly elevated correlation with stroke risk across the study population (Figure 3). The findings indicate that the impact of WWI on the risk of incident stroke may be related to age, smoking status, marital status, and the presence of hypertension (Figure 3).

### Sensitivity analysis

This research utilized Cox regression analysis to assess the association between WWI and stroke risk, excluding datasets with missing values (Supplementary Table 1). Both unadjusted and adjusted models showed a notable link association between WWI and stroke risk, which remained robust even after controlling for multiple confounding factors (Supplementary Table 2). Consistent with the findings in Table 2, the analysis confirmed that the imputation of missing data did not substantially alter the results, suggesting that the multiple imputation approach effectively addressed missing data issues and further validated the reliability of the study’s outcomes. A sensitivity analysis, which excluded stroke cases identified during the first 2 years of follow-up, continued to demonstrate a significant link between higher WWI levels and increased stroke risk. These results highlight the potential utility of WWI as a predictive indicator for stroke, with its predictive capacity remaining stable even after the exclusion of early stroke events (Supplementary Table 3).

### The predictive value of WWI for stroke risk

**Figure 4:**
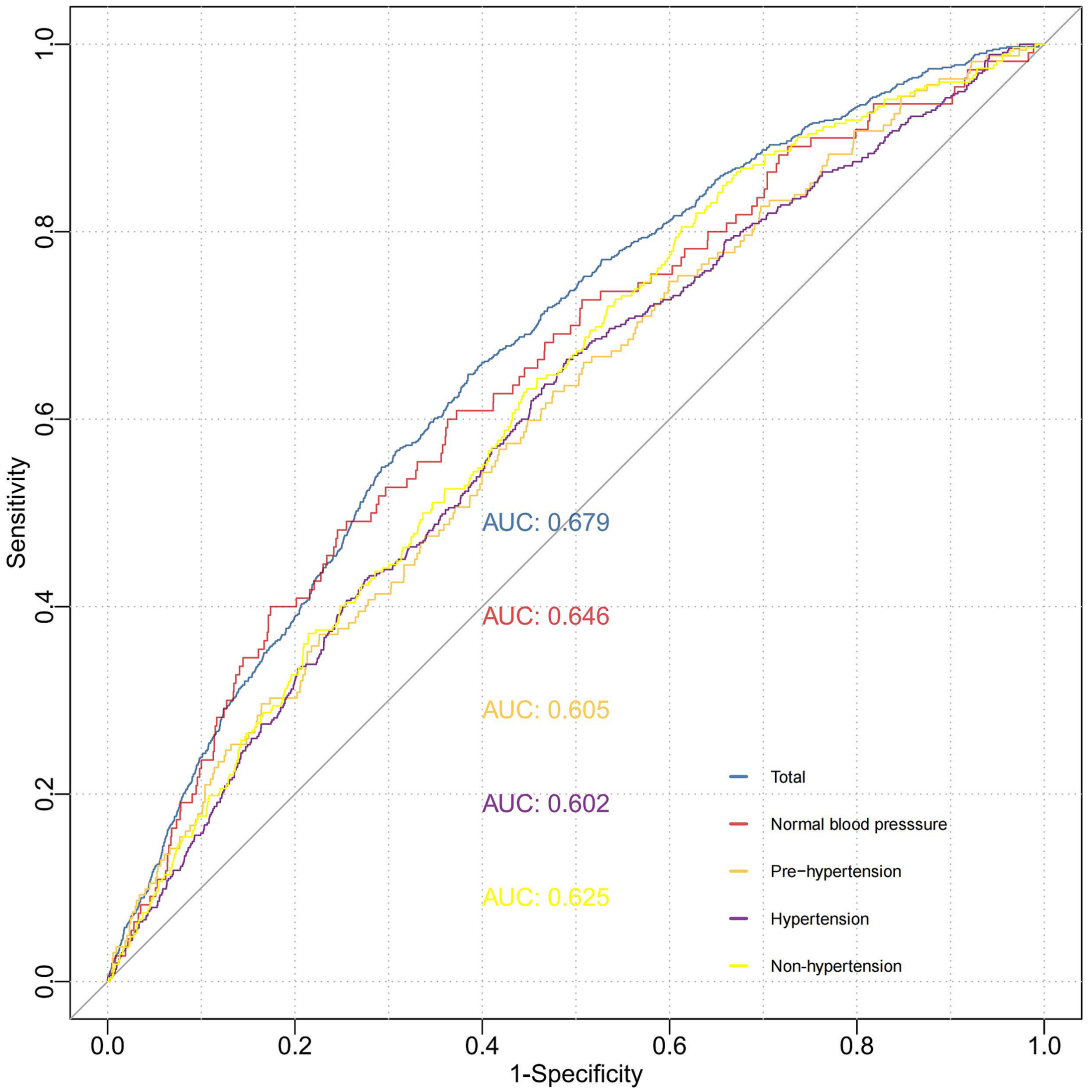
ROC curve analysis to evaluate the predictive performance of different anthropometric indicators on stroke risk. Model 1: Unadjusted. Model 2: Adjusted for age, gender, education level, marriage status, drinking status, smoking status. Model 3: Model 2 with additional adjustment for creatinine, BUN, eGFR, diabetes mellitus and dyslipidemia. WWI, weight-adjusted waist index; BUN, blood urea nitrogen; eGFR, estimated glomerular filtration rate; BMI, body mass index; WC, waist circumference; WHR, waist-to-hip ratio; ROC, receiver operating characteristic curve; AUC, area under the curve.

**Figure 5:**
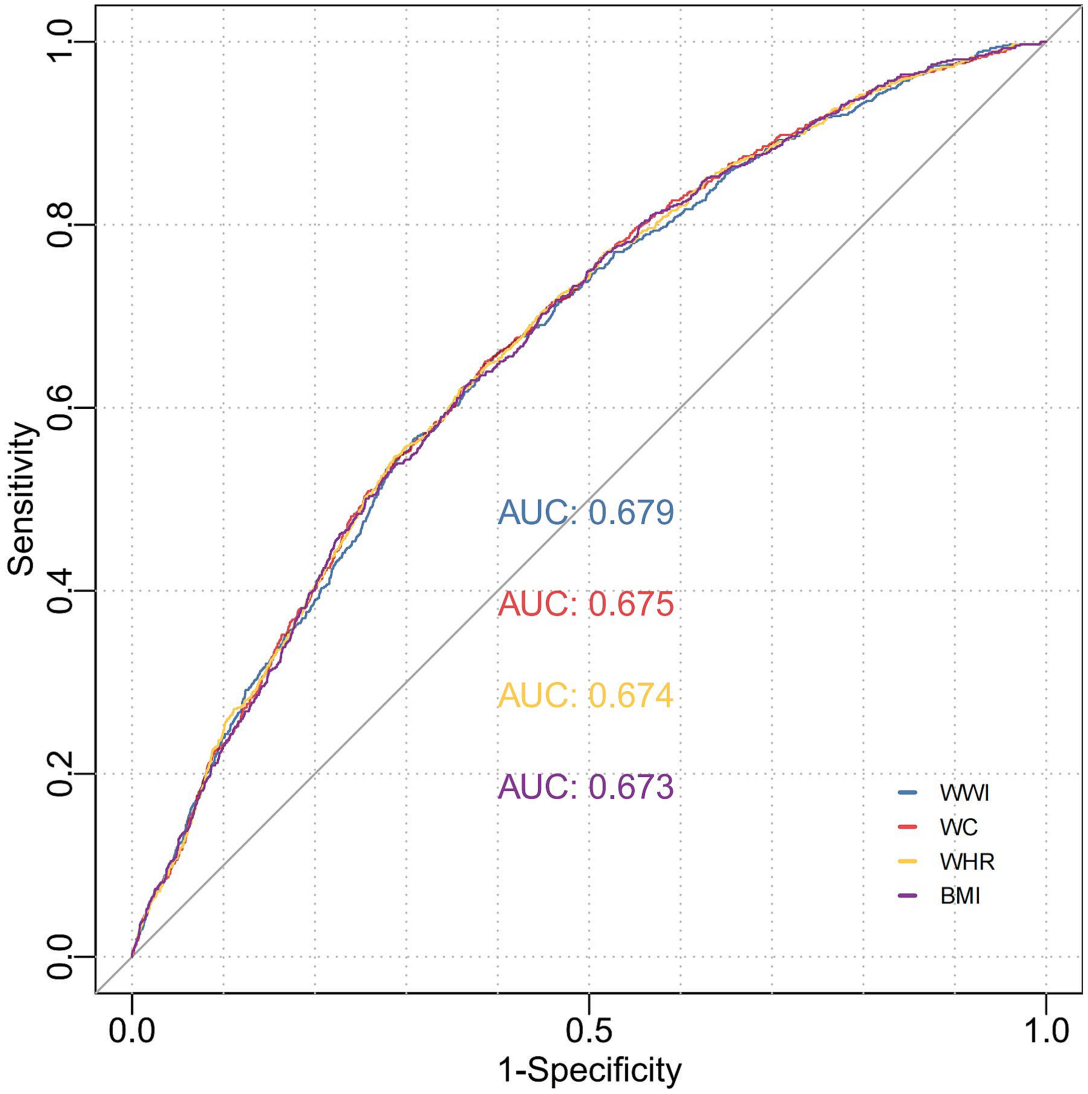
ROC curve analysis to evaluate the predictive performance of WWI for stroke across different blood pressure states. Model 1: Unadjusted. Model 2: Adjusted for age, gender, education level, marriage status, drinking status, smoking status. Model 3: Model 2 with additional adjustment for creatinine, BUN, eGFR, diabetes mellitus, dyslipidemia, and hypertension. WWI, weight-adjusted waist index; BUN, blood urea nitrogen; eGFR, estimated glomerular filtration rate; BMI, body mass index; WC, waist circumference; WHR, waist-to-hip ratio; ROC, receiver operating characteristic curve; AUC, area under the curve.

BMI and body weight are widely recognized as fundamental anthropometric parameters, whereas central obesity is typically evaluated using WC and the WHR. To assess the predictive efficacy of WWI in comparison to these conventional anthropometric measures for stroke risk, we calculated the AUC. Our analysis revealed that WWI exhibited superior predictive performance compared to the four traditional anthropometric indicators, with an AUC of 0.679 (95%CI, 0.651–0.721, all *p* < 0.001) (Figure 4 and Supplementary Table 4). Specifically, WWI demonstrated the highest discriminative ability, achieving statistically significant AUC values (*p* < 0.001) for stroke prediction across pre-hypertensive (AUC = 0.605, 95% CI: 0.561–0.649), hypertensive (AUC = 0.672, 95%CI: 0.575–0.759), non-hypertensive (AUC = 0.625, 95%CI: 0.593–0.656) and normal blood pressure individuals (AUC = 0.646, 95%CI: 0.583–0.699). Notably, WWI surpassed traditional metrics in accurately predicting stroke incidence, particularly within hypertensive populations (Figure 5 and Supplementary Table 4).

### Mediation effect analysis of hypertension

**Figure 6:**
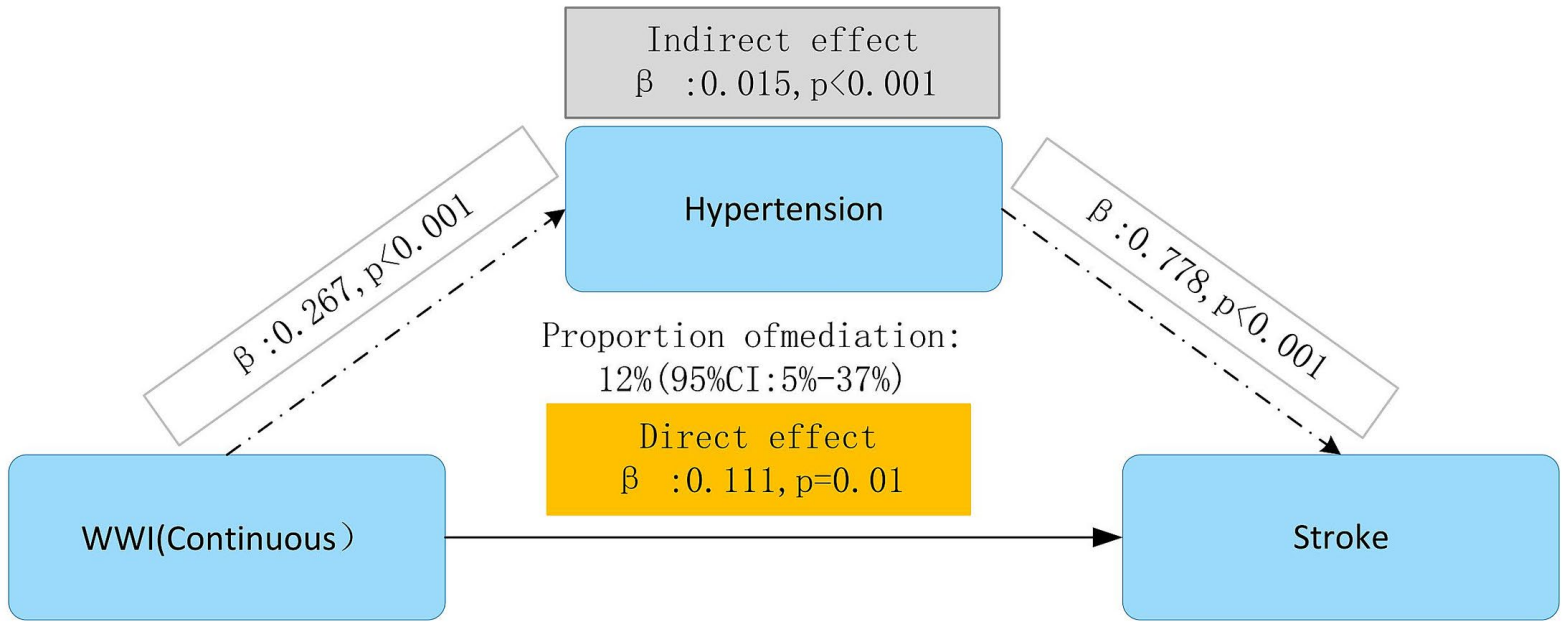
Mediation effect analysis of hypertension in the association between WWI and stroke. Model 1: Unadjusted. Model 2: Adjusted for age, gender, education level, marriage status, drinking status, smoking status. Model 3: Model 2 with additional adjustment for creatinine, BUN, eGFR, diabetes mellitus, dyslipidemia, and hypertension. WWI, weight-adjusted waist index; BUN, blood urea nitrogen; eGFR, estimated glomerular filtration rate; HR, hazard ratio, CI, confidence interval.

In analyzing a hypothesized pathway through mediation, the direct association between WWI and stroke continued to be statistically significant (*β* = 0.111, *p* = 0.01), suggesting that greater WWI levels might be related to a heightened stroke risk. There was also a modest but statistically significant indirect effect observed through hypertension (*β* = 0.015, *p* < 0.001), which accounted for about 12% of the total effect (95%CI: 5–37%). The findings suggest that within the model’s constraints, hypertension might partially elucidate the relationship between WWI and stroke. Significantly, hypertension itself was strongly and significantly associated with stroke (*β* = 0.778, *p* < 0.001), confirming its established importance as a key cardiovascular risk factor (Figure 6).

## Discussion

Our longitudinal analysis utilizing CHARLS data revealed a significant association between WWI and subsequent stroke risk among middle-aged and elderly populations, with particularly pronounced associations observed across varying blood pressure statuses. Notably, the analysis demonstrated that elevated WWI values exhibited a stronger correlation with increased stroke risk in pre-hypertensive and hypertensive individuals compared to their normal blood pressure. Moreover, subgroup analysis revealed a stronger link between WWI and stroke risk among participants under the age of 60 (*p* < 0.001). In addition, WWI showed a stronger relationship to stroke risk than BMI, WC, and WHR. This differential association suggests that WWI may serve as a robust and reliable predictive indicator of stroke risk across diverse metabolic profiles. Mediation effect analysis indicated that hypertension partially mediated the impact of WWI on stroke (*β*: 0.015, *p* < 0.001), accounting for 12% of WWI’s total effect on stroke incidence (Given that WWI and hypertension were recorded at the same baseline point, the direction and timing of this mediation pathway cannot be clearly defined. Therefore, these findings should be approached with caution and seen as exploratory instead of confirmatory). These findings underscore the clinical utility of WWI as an anthropometric biomarker for assessing body composition and central adiposity in stroke risk stratification, particularly among individuals with blood pressure abnormalities.

Obesity is a chronic disease resulting from prolonged energy intake surpassing energy expenditure, leading to excessive accumulation of body fat. It not only affects physical appearance but is also closely associated with various health issues, particularly cardiovascular diseases (23). BMI and WC are standard metrics for assessing obesity. Research conducted on 21,000 men in the US demonstrated that every unit increase in BMI correlated with a 6% elevated risk of total, ischemic, and hemorrhagic stroke (24). A cross-sectional investigation carried out by Cho et al., which included 21,749,261 Korean participants, found a direct linear association between waist circumference (WC) and the occurrence of ischemic stroke (25). Despite increasing evidence connecting these conventional obesity measures to stroke, the obesity paradox remains unresolved (26). A key limitation of these traditional indicators is their failure to distinguish between fat mass and muscle mass (27). For instance, BMI cannot differentiate between weight increases caused by high levels of lean body mass and those caused by fat mass. WWI is a straightforward anthropometric measure derived from the formula [ln(WC) = *β*₀ + β₁ ln(weight) + *ε*], which standardizes WC for weight using least squares regression of log-transformed WC on log-transformed weight (28). Thus, WWI integrates the advantages of WC while minimizing its association with BMI, allowing it to evaluate both body fat and muscle tissue. WWI is suitable for diverse ethnic groups and populations, potentially providing enhanced consistency and dependability, especially in cross-ethnic or multi-center research (29). A large-scale prospective cohort study, including 12,447 Chinese participants, found a significant association between elevated WWI levels and an increased risk of heart-related incidents and overall mortality (30). This result is additionally corroborated by the study conducted by Cai et al. (31), which revealed a significant relationship between WWI and the risk of all-cause overall mortality. Despite these well-documented links, the connection between WWI and stroke continues a topic of debate. A study involving 23,389 individuals in China suggests a potential connection between stroke and WWI. The findings reveal that an elevated WWI is linked to an increased risk of stroke (32). In a separate cross-sectional analysis focusing on American adults, increased WWI levels were found to be strongly associated with a greater risk of cardiovascular disease. However, after accounting for potential confounding factors, the assessment pointed to no significant connection between WWI and stroke (33). The outcomes of these two studies exhibit both inconsistency and a cross-sectional nature. Compared to previous studies, our study is a longitudinal cohort study in which we demonstrated the association between WWI levels and stroke occurrence among middle-aged and older adults across different blood pressure status. Although this is not the first time the field has been studied based on previous literature, the novelty of this study lies in its exploration of hypertension as a mediator in the association between the WWI and the risk of stroke. Based on the observed differences in the association between WWI and stroke risk across blood pressure categories, we theorized that hypertension might partially explain this relationship. This hypothesis aligns with the established pathophysiological role of central obesity in elevating blood pressure, glucose, and lipid levels. By employing mediation analysis, we formally tested this hypothesis and found that hypertension partially mediated the connection between WWI and stroke risk, shedding light on the underlying mechanisms. Furthermore, we compared WWI with conventional anthropometric measures, including BMI, WC, and WHR, and demonstrated that WWI exhibited superior predictive value for stroke risk.

Our research validated the primary hypothesis that elevated WWI levels show a strong correlation with a higher likelihood of stroke, especially in individuals with abnormal blood pressure levels. By examining WWI as both categorical and continuous variables about stroke risk, we gained a deeper and more detailed insight into this relationship. This dual analytical approach enabled us to assess stroke risk at different WWI thresholds more accurately. The results revealed a clear dose–response pattern, showing that stroke risk rises steadily as WWI increases. Notably, this relationship persisted even after controlling for likely confounding elements, emphasizing the enduring influence of elevated WWI on cerebrovascular health. Subgroup analyses targeting participants below 60 years of age further validated the stability of this relationship across varied demographic groups. To mitigate the possibility of reverse causation, sensitivity analyses were performed by removing stroke incidents recorded within the initial 2 years of follow-up. Furthermore, advanced data imputation methods were utilized to enhance the reliability and robustness of the findings. Cox proportional hazards models consistently highlighted a clear relationship between elevated WWI levels and heightened stroke risk, even after the exclusion of early-onset stroke cases. The results demonstrate the possible advantages of WWI as a robust and independent indicator for assessing stroke risk. The study further employed ROC analysis to evaluate the predictive performance of WWI against conventional anthropometric including BMI, WC, and WHR-in forecasting stroke incidence. Analytical results revealed WWI’s superior prognostic utility, as evidenced by its significantly larger AUC compared to other metrics. In addition to the AUC values, a deeper examination of diagnostic performance indicators reveals that WWI offers advantages over traditional anthropometric measures in certain respects. WWI demonstrated a sensitivity of 0.648 and an overall accuracy of 0.617, which were slightly higher or comparable to WC, WHR, and BMI, indicating superior or equivalent performance in identifying stroke risk. Significantly, the PLR of 1.683 and NLR of 0.572 for WWI suggest stable predictive performance in diverse clinical situations. WWI’s heightened sensitivity in clinical practice enhances its role in screening and risk stratification by lowering the chances of missing cases. The stable PLR and NLR across risk thresholds further support its consistency and potential utility in diverse clinical settings. Furthermore, WWI had a PPV of 0.094 and an NPV of 0.966, indicating its proficiency in accurately detecting non-stroke cases and minimizing false negatives. In addition, the PPA stood at 0.648, while the NPA was 0.615, reflecting WWI’s balanced effectiveness in classifying both positive and negative cases accurately. The TPA of 0.617 and kappa coefficient of 0.070 for WWI show moderate agreement between predicted and actual stroke cases, pointing to its clinical relevance in risk stratification. It is worth noting that the ROC curve comparison validates the enhanced predictive power of WWI, especially under different blood pressure conditions. These observations underscore WWI’s clinical significance as a stratification tool for stroke risk detection, particularly in hypertensive populations of individuals aged middle years and beyond. This study holds significant clinical implications. For individuals with elevated WWI levels, early blood pressure assessment and timely intervention could effectively mitigate stroke risk and enhance clinical outcomes.

Several underlying mechanisms may account for the evident link between WWI and stroke. Firstly, a rise in WWI indicates excessive fat accumulation and a reduction in muscle mass. This imbalance between fat and muscle disrupts the normal release of adipocyte cytokines, induces inflammatory responses, impairs endothelial function, and diminishes physical activity. Moreover, individuals with hypertension often experience hemodynamic irregularities, such as blood pressure variability and heightened vascular resistance, which further exacerbate endothelial dysfunction and arteriosclerosis. The combination of these factors ultimately contributes to the onset of cardiovascular and cerebrovascular diseases (34, 35). Secondly, WWI is related to the imbalance of the intestinal microbiome, each of these elements will speed up the development of atherosclerosis, leading to a higher likelihood of stroke (36, 37). Thirdly, obesity exhibits a pathological and physiological clustering of multiple diseases. The typical manifestation involves insulin resistance, which is the main driving factor of atherosclerotic metabolic disorder caused by the imbalance of the IRS-1/PI3K/Akt pathway. It is worth noting that 78% of hypertensive patients will develop metabolic syndrome. This synergistic metabolism increases the risk of stroke by 1.7 times by accelerating the formation of carotid plaques and uncoupling of endothelial nitric oxide synthase (38). Fourthly, obesity can trigger neuroendocrine alterations and activate the sympathetic nervous system, potentially resulting in excessive stimulation of the renin-angiotensin system. These physiological responses may further exacerbate vascular injury and induce vasoconstriction, contributing to the development of stroke (39). Fifthly, obstructive sleep apnea (OSA) has been identified as a significant risk factor for cardiovascular and cerebrovascular diseases, as well as a prevalent complication among obese individuals (40). This underlying mechanism helps explain our findings, particularly the stronger link between WWI and stroke risk among individuals with hypertension. In contrast, individuals with normal blood pressure generally exhibit a lower overall risk of stroke, where the influence of WWI may be less pronounced or overshadowed by other contributing factors. These factors may include gender, alcohol consumption, diabetes, dyslipidemia, and other metabolic or lifestyle variables.

### Strengths and limitations

Several strengths stand out in this study. Firstly, we analyzed data from a representative group of middle-aged and elderly individuals from CHARLS, which adheres to stringent research protocols and quality control standards, thereby ensuring the reliability and generalizability of our findings. Secondly, we performed extensive subgroup analyses to better understand the tie between WWI and stroke across a range of demographic and clinical subgroups. Thirdly, we accounted for a wide range of potential confounders to reduce their influence and strengthen the validity of our results. Fourthly, the longitudinal design of the study allowed us to explore the temporal relationship between WWI and stroke risk over time. Nevertheless, certain limitations must be acknowledged. Firstly, although we adjusted for numerous confounding variables, the potential for residual confounding remains, including factors such as dietary habits that may affect both obesity and stroke risk, as well as genetic influences. Secondly, the duration of the study might not have been sufficient to fully assess the long-term link between WWI and stroke. Thirdly, during data analysis, we excluded participants who did not meet the inclusion criteria. Starting from an initial sample of 17,705 participants, we applied rigorous screening to ensure the quality and reliability of the data, ultimately including 12,580 participants in the final analysis. Although these exclusions were necessary to improve the internal validity of the study and enhance the accuracy of the analyses, we acknowledge that this process may have reduced the sample size and potentially limited the statistical power to detect subtle associations. Finally, the study’s cross-sectional measurement setup constrained the mediation analysis regarding hypertension. WWI and hypertension were evaluated at the same time initially, making it difficult to determine their chronological sequence. In the absence of repeated measurements, the theoretical causal pathway in which WWI influences stroke risk through hypertension cannot be fully substantiated by empirical evidence. Therefore, these findings should be approached with caution and seen as exploratory instead of confirmatory.

## Conclusion

The outcomes reveal a strong positive relationship between increased WWI and stroke risk in elderly hypertensive populations. By employing a longitudinal design, we were able to dynamically examine the temporal interaction between WWI and the progression of stroke. The strong predictive value of WWI underscores its clinical relevance for assessing stroke risk, guiding early intervention measures, and reducing stroke incidence. Future research should strive to incorporate a wider variety of confounding variables and more diverse populations to further clarify the pathways through which WWI influences stroke risk. Such efforts could offer critical insights for refining and enhancing stroke prevention strategies.

## Data Availability

The datasets presented in this study can be found in online repositories. The names of the repository/repositories and accession number(s) can be found in the article/Supplementary material.

## Footnotes

^1^ http://charls.pku.edu.cn
