# Supplementary material for: Hypertension mediates the association between weight-adjusted waist index and new onset stroke risk in middle-aged and older Chinese adults: evidence from the CHARLS study
Source: Front Neurol. 2025 Jul 11;16:1587176. doi: 10.3389/fneur.2025.1587176 (PMC12289689; doi:10.3389/fneur.2025.1587176)
Supplement: Supplementary file 1 [file Table_1.DOCX]

**Supplementary Table 1** Distribution of missing data

| **Characteristic** | **No. of missing values** | **Percent (%)** |
| --- | --- | --- |
| **BMI** | 109 | 0.84 |
| **WWI** | 0 | 0.00 |
| **Age** | 0 | 0.00 |
| **Gender** | 9 | 0.11 |
| **SBP** | 143 | 1.79 |
| **DBP** | 161 | 2.02 |
| **Marriage status** | 0 | 0.00 |
| **Education level** | 6 | 0.08 |
| **Smoking status** | 60 | 0.75 |
| **Drinking status** | 9 | 0.11 |
| **Heart rate** | 105 | 1.32 |
| **Diabetes mellitus** | 23 | 0.29 |
| **Hypertension** | 0 | 0.00 |
| **Dyslipidemia** | 156 | 1.96 |
| **HbA1c** | 4522 | 56.74 |
| **FBG** | 4603 | 57.76 |
| **BUN** | 4585 | 57.54 |
| **eGFR** | 4614 | 57.90 |
| **Stroke** | 0 | 0.00 |

**Abbreviations**: WWI, weight-adjusted waist index; SBP, systolic blood pressure; DBP, diastolic blood Pressure; BMI, body mass index; WC, waist circumference; WHR, waist-to-hip ratio; FBG, fasting blood glucose; BUN, blood urea nitrogen; eGFR, Estimated Glomerular Filtration Rate; HbA1c, hemoglobin A1c.

**Supplementary Table 2** The stroke incidence based on WWI across the three models with missing data

| **Characteristic** | **N** | **Event N** | **Model 1** | | | | **Model 2** | | | | **Model 3** | | | | |
| --- | --- | --- | --- | --- | --- | --- | --- | --- | --- | --- | --- | --- | --- | --- | --- |
|  |  |  | **HR** | **95% CI** | | **p-value** | **HR** | **95% CI** | | **p-value** | **HR** | **95% CI** | | **p-value** | |
| **Total** | | | | | | | | | | | | | | | |
| **Continues-WWI** | 12,580 | 510 | 1.27 | 1.18, 1.37 | <0.001 | | 1.21 | | 1.11, 1.32 | <0.001 | 1.12 | | 1.01, 1.24 | | 0.039 |
| **WWI (Quartile)** | 12,580 | 510 |  |  |  | |  | |  |  |  | |  | |  |
| **Q1** | 3,165 |  | Reference | Reference |  | | Reference | | Reference |  | Reference | | Reference | |  |
| **Q2** | 3,171 |  | 1.34 | 1.07, 1.67 | 0.011 | | 1.30 | | 1.04, 1.63 | 0.023 | 1.28 | | 0.98, 1.68 | | 0.073 |
| **Q3** | 3,146 |  | 1.41 | 1.13, 1.77 | 0.002 | | 1.34 | | 1.07, 1.68 | 0.012 | 1.25 | | 0.95, 1.64 | | 0.114 |
| **Q4** | 3,098 |  | 1.76 | 1.43, 2.18 | <0.001 | | 1.53 | | 1.20, 1.95 | <0.001 | 1.30 | | 0.97, 1.74 | | 0.083 |

**Model 1**: Unadjusted

**Model 2**: Adjusted for age, gender, education level, marriage status, drinking status, smoking status

**Model 3**: Model 2 with additional adjustment for creatinine, BUN, eGFR, diabetes mellitus, dyslipidemia, and hypertension

**Abbreviation: Abbreviation:** WWI, weight-adjusted waist index; BUN, blood urea nitrogen; eGFR, estimated glomerular filtration rate; HR, hazard ratio, CI, confidence interval

**Supplementary Table 3** The stroke incidence based on WWI across the three models and excluding strokes occurring within the first two years

| **Characteristic** | **N** | **Event N** | **Model 1** | | | | **Model 2** | | | | **Model 3** | | | | |
| --- | --- | --- | --- | --- | --- | --- | --- | --- | --- | --- | --- | --- | --- | --- | --- |
|  |  |  | **HR** | **95% CI** | | **p-value** | **HR** | **95% CI** | | **p-value** | **HR** | **95% CI** | | **p-value** | |
| **Total** | | | | | | | | | | | | | | | |
| **Continues-WWI** | 12,401 | 548 | 1.22 | 1.12, 1.34 | <0.001 | | 1.15 | | 1.04, 1.27 | 0.006 | 1.08 | | 0.98, 1.20 | | 0.018 |
| **WWI (Quartile)** | 12,401 | 548 |  |  |  | |  | |  |  |  | |  | |  |
| **Q1** | 3,130 |  | Reference | Reference |  | | Reference | | Reference |  | Reference | | Reference | |  |
| **Q2** | 3,127 |  | 1.37 | 1.05, 1.77 | 0.019 | | 1.34 | | 1.03, 1.74 | 0.028 | 1.24 | | 0.95, 1.61 | | 0.111 |
| **Q3** | 3,103 |  | 1.48 | 1.14, 1.91 | 0.003 | | 1.40 | | 1.07, 1.82 | 0.013 | 1.23 | | 0.94, 1.60 | | 0.130 |
| **Q4** | 3,041 |  | 1.80 | 1.40, 2.30 | <0.001 | | 1.59 | | 1.20, 2.10 | 0.001 | 1.33 | | 1.00, 1.75 | | 0.048 |

**Model 1**: Unadjusted

**Model 2**: Adjusted for age, gender, education level, marriage status, drinking status, smoking status

**Model 3**: Model 2 with additional adjustment for creatinine, BUN, eGFR, diabetes mellitus, dyslipidemia, and hypertension

**Abbreviation:** WWI, weight-adjusted waist index; BUN, blood urea nitrogen; eGFR, estimated glomerular filtration rate; HR, hazard ratio, CI, confidence interval

**Supplementary Table 4** ROC curves of anthropometric measures in predicting stroke risk

| **Characteristic** | **Cutoff** | **AUC (95%CI)** | **AUC.SE** | **ACC** | **SEN** | **SPE** | **PLR** | **NLR** | **PPV** | **NPV** | **PPA** | **NPA** | **TPA** | **KAPPA** | **P-value** |
| --- | --- | --- | --- | --- | --- | --- | --- | --- | --- | --- | --- | --- | --- | --- | --- |
| Total | 0.487 | 0.679  (0.651, 0.721) | 0.010 | 0.617 | 0.648 | 0.615 | 1.683 | 0.572 | 0.094 | 0.966 | 0.648 | 0.615 | 0.617 | 0.070 | P<0.001 |
| Normal blood pressure | 0.232 | 0.646  (0.593, 0.699) | 0.027 | 0.636 | 0.600 | 0.637 | 1.653 | 0.628 | 0.046 | 0.982 | 0.600 | 0.637 | 0.636 | 0.035 | P<0.001 |
| Pre-hypertension | 0.137 | 0.605  (0.561, 0.649) | 0.022 | 0.529 | 0.630 | 0.525 | 1.324 | 0.706 | 0.058 | 0.968 | 0.630 | 0.525 | 0.529 | 0.027 | P<0.001 |
| Hypertension | -0.039 | 0.602  (0.575, 0.759) | 0.014 | 0.524 | 0.664 | 0.510 | 1.355 | 0.659 | 0.118 | 0.939 | 0.664 | 0.510 | 0.524 | 0.056 | P<0.001 |
| Non-hypertension | -0.074 | 0.625  (0.593, 0.656) | 0.016 | 0.362 | 0.849 | 0.343 | 1.293 | 0.439 | 0.046 | 0.984 | 0.849 | 0.343 | 0.362 | 0.021 | P<0.001 |
| **Characteristic** | Cutoff | AUC | AUC.SE | ACC | SEN | SPE | PLR | NLR | PPV | NPV | PPA | NPA | TPA | KAPPA | P-value |
| WWI | 0.487 | 0.679  (0.651, 0.721) | 0.010 | 0.617 | 0.648 | 0.615 | 1.683 | 0.572 | 0.094 | 0.966 | 0.648 | 0.615 | 0.617 | 0.070 | P<0.001 |
| WC | 0.484 | 0.675  (0.655, 0.694) | 0.010 | 0.616 | 0.651 | 0.614 | 1.685 | 0.569 | 0.094 | 0.966 | 0.651 | 0.614 | 0.616 | 0.070 | P<0.001 |
| WHR | 0.514 | 0.674  (0.655, 0.693) | 0.010 | 0.640 | 0.620 | 0.641 | 1.730 | 0.592 | 0.096 | 0.965 | 0.620 | 0.641 | 0.640 | 0.073 | P<0.001 |
| BMI | 0.540 | 0.673  (0.653, 0.692) | 0.010 | 0.630 | 0.630 | 0.630 | 1.701 | 0.588 | 0.094 | 0.965 | 0.630 | 0.630 | 0.630 | 0.071 | P<0.001 |

**Abbreviation:** WWI, weight-adjusted waist index; BUN, blood urea nitrogen; eGFR, estimated glomerular filtration rate; BMI, body mass index; WC, waist circumference; WHR, waist-to-hip Ratio; ROC, receiver operating characteristic curve; AUC, area under the curve; SE, standard errors; ACC, overall accuracy; SEN, sensitivity; SPE,specificity; PLR, positive likelihood ratio; NLR,negative likelihood ratio; PPV, positive predictive value; NPV,negative predictive value; PPA,positive predictive agreement; NPA,negative predictive agreement; TPA,total predictive agreement; KAPPA, kappa statistic

**Model 1**: Unadjusted

**Model 2**: Adjusted for age, gender, education level, marriage status, drinking status, smoking status

**Model 3**: Model 2 with additional adjustment for creatinine, BUN, eGFR, diabetes mellitus, dyslipidemia, and hypertension
